# The effect of hand dominance on results in the Functional Head Impulse Test

**DOI:** 10.1017/S0022215126105258

**Published:** 2026-08

**Authors:** Gülçin Hançer Arslan, Ayşenur Kanık, Aysu Ünlü, Orkun Tahir Aran, Mine Baydan Aran

**Affiliations:** 1 Department of Audiometry, Vocational School of Health Services, Trakya University, Edirne, Türkiye; 2 Department of Audiology, Faculty of Health Sciences, Bezmialem Vakif University, Istanbul, Türkiye; 3 Department of Audiology, Institute of Graduate Studies, Istanbul University-Cerrahpasa, Istanbul, Türkiye; 4 Department of Audiology, Faculty of Health Sciences, Trakya University, Edirne, Türkiye; 5 Department of Occupational Therapy, Faculty of Health Sciences, Hacettepe University, Ankara, Türkiye; 6 Department of Audiology, Faculty of Health Sciences, Ankara University, Ankara, Türkiye

**Keywords:** Vestibular Function Tests, head impulse test, functional laterality, reflex, vestibulo-ocular

## Abstract

**Objective:**

The vestibulo-ocular reflex is essential for maintaining visual stability during head movements. Individual neurophysiological characteristics, such as hand dominance, may influence vestibular responses through hemispheric lateralisation. This study aimed to investigate the effect of hand dominance on functional vestibulo-ocular reflex performance using the Functional Head Impulse Test.

**Methods:**

Fifty-eight healthy volunteers were classified as right- or left-handed using the Edinburgh Handedness Inventory. Functional Head Impulse Test reading scores were obtained during passive rightward and leftward head impulses at acceleration levels of 4000, 5000 and 6000°/s^2^.

**Results:**

No significant differences were observed between right- and left-handed individuals during rightward head movements. In contrast, during leftward head movements, right-handed individuals demonstrated significantly higher Functional Head Impulse Test scores at 4000 and 6000°/s^2^, as well as higher total reading scores.

**Conclusion:**

Hand dominance influences functional vestibulo-ocular performance, particularly during leftward head impulses. Considering hand dominance in the interpretation of Functional Head Impulse Test results may enhance the accuracy and individualisation of vestibular assessments.

## Introduction

The vestibular system plays a vital role in maintaining balance, postural control and visual stability by continuously providing the central nervous system with information about the head’s position and movements in space.^1^ Angular accelerations are detected by the semicircular canals, while linear accelerations are sensed by the otolith organs.^2^ One of the most important reflex mechanisms developed to prevent retinal image slip during sudden head movements is the vestibulo-ocular reflex.^3^ This reflex ensures visual stabilisation by generating compensatory eye movements in the opposite direction to head motion, thereby maintaining clear vision during dynamic activities.^4^

Although the Head Impulse Test is widely used in the clinical evaluation of the vestibulo-ocular reflex, its qualitative nature and dependence on examiner experience limit its objectivity and reproducibility.^5^ To overcome these limitations, the Functional Head Impulse Test was developed as a quantitative method that measures the percentage of optotypes correctly identified during passive head impulses applied at specific angular accelerations. The Functional Head Impulse Test provides a functional assessment of gaze stabilisation under dynamic conditions and has gained increasing clinical relevance as a result of its speed, reliability and ecological validity.^6^^,^^7^

Hand dominance refers to the preferential use of one hand for motor and sensory activities, and reflects functional asymmetry between the cerebral hemispheres. Globally, left-handedness is reported in approximately 10.6 per cent of the population,^8^ while studies conducted in Turkey have shown a left-handedness prevalence of around 20.3 per cent.^9^ Handedness is considered a key indicator of hemispheric lateralisation of motor, cognitive and sensory functions, therefore it may influence not only motor performance but also the processing of vestibular information.^10^

Neuroimaging and behavioural studies have demonstrated that vestibular inputs are predominantly processed in the right cerebral hemisphere, regardless of handedness.^11^^,^^12^ Meta-analytical and functional connectivity studies further indicate that the vestibular cortical network exhibits hemispheric asymmetry, with the right hemisphere playing a dominant role in spatial orientation and self-motion perception.^13^ This hemispheric specialisation suggests that vestibular responses may vary depending on both the direction of the stimulus and individual lateralisation patterns.

Despite growing evidence on cortical vestibular lateralisation, the functional impact of hand dominance on dynamic vestibulo-ocular performance has not been systematically investigated using the Functional Head Impulse Test. Previous studies have primarily focused on hemispheric activation patterns or static vestibular measures, whereas limited data are available on how handedness influences functional gaze stabilisation during high-acceleration head movements. The relationship between hand dominance and Functional Head Impulse Test performance remains insufficiently understood.

The interaction between stimulus direction and hemispheric dominance is also thought to play a role in vestibular processing. Previous research suggests that vestibular responses may differ depending on whether head movements preferentially stimulate the dominant or non-dominant hemisphere.^11^^–^^13^ Evaluating head movement direction alongside hand dominance may provide further insight into the functional organisation of the vestibular system.

In this study, the effect of hand dominance on vestibulo-ocular reflex responses was evaluated using the Functional Head Impulse Test during rightward and leftward head movements at different acceleration levels. The findings aim to clarify the relationship between handedness, hemispheric lateralisation and functional vestibular performance, and to contribute to more individualised interpretation of vestibular test results in clinical practice.

## Methods

### Individuals

The aim of this study was to investigate the effect of brain lateralisation associated with hand dominance on the vestibulo-ocular system and gaze stabilisation using the Functional Head Impulse Test.

A total of 58 healthy volunteers were included in the study. Participants were required to meet the following inclusion criteria: (1) no history of vestibular disorders; (2) no current or past head or neck conditions that could interfere with the test procedure; (3) no uncorrected refractive errors (corrected vision with glasses or contact lenses was acceptable); (4) no diagnosis of migraine or vestibular migraine; and (5) no current vestibular complaints.

All participants were informed about the study procedures and written informed consent was obtained prior to participation. The study was conducted in accordance with the 1975 Helsinki Declaration (as revised in 2008) and received ethical approval from the Human Research Ethics Committee of Ankara University Faculty of Medicine. The authors declare that all procedures contributing to this study complied with the ethical standards of the relevant national and institutional guidelines (Human Research Ethics Committee of Ankara University Faculty of Medicine/Ethics Committee Approval Date: 15.09.2025, Decision No: İ07-703-25).

### Assessments

#### Edinburgh Handedness Questionnaire

Hand dominance was determined using the Edinburgh Handedness Questionnaire, originally developed by Oldfield (1971)^14^ and validated for the Turkish population in 2019.^9^ The questionnaire assesses hand preference for 10 common daily activities, including writing, brushing teeth, using a spoon, using scissors, using a hammer, lighting a match, threading a needle, kicking a soccer ball, looking through a telescope and opening a box lid.

For the purposes of this study, each activity was scored as −1 for left-hand preference and +1 for right-hand preference. A lateralisation index was calculated for each participant based on these scores. Only individuals with clear right-hand dominance or clear left-hand dominance were included in the study, while those with mixed or ambidextrous hand preference were excluded to ensure distinct lateralisation groups.

#### Functional Head Impulse Test

After confirming that the participants met the inclusion criteria, the test protocol was explained in detail. The Functional Head Impulse Test (Functional Head Impulse Test 1.0; Beon Solutions, Zero Branco (TV), Italy) was administered in the lateral plane using an optotype presentation duration of 60 ms.

Participants were seated 1.5 m in front of the computer screen, aligned with the centre of the display. An accelerometer headband was placed on the participant’s head to monitor head movement parameters. The Landolt C optotype was used, with the gap orientation presented in 8 possible directions at 45° intervals.

First, static visual acuity was assessed. Participants were instructed to identify the orientation of the optotype while keeping their head stationary. The optotype size was progressively reduced after each correct response and was defined as the LogMAR level at which the participant made three consecutive errors or achieved a correct response at 0.0 LogMAR.

For the dynamic phase of the test, the optotype size was automatically set by the software by adding 0.6 LogMAR to the participant’s individual static visual acuity threshold. A short familiarisation trial was performed to allow participants to become accustomed to the response keyboard and data from this trial were excluded from the analysis.

During the test, participants were instructed to maintain fixation on a grey dot at the centre of the screen while passive head impulses were applied in the lateral plane at approximately 15–20° to the right and left of the midline. Head impulses were delivered manually by an experienced examiner following standardised Functional Head Impulse Test procedures to ensure consistent amplitude and acceleration profiles. The head movements were performed at acceleration levels of 4000, 5000 and 6000°/s^2^.

A total of 30 valid head impulses were recorded for each participant, with 10 impulses delivered at each acceleration level. The software automatically excluded trials with inadequate acceleration or incorrect timing of optotype presentation. The percentage of correctly identified optotypes was calculated for each acceleration level and for the total number of impulses. Each participant’s percentage of correctly identified optotypes was automatically compared with the built-in normative reference values provided by the Beon Solutions Functional Head Impulse Test software, which simultaneously computes a Z-based statistical confidence index and flags any deviation exceeding its predefined threshold as performance falling outside the normal range.

Reading scores obtained during rightward and leftward head movements were analysed separately. These scores were then compared according to hand dominance and head movement direction to evaluate potential lateralisation effects on functional vestibulo-ocular performance.

### Statistical analysis

Statistical analyses were performed using SPSS Statistics version 23.0 (IBM Corp., Armonk, NY, USA). The distribution characteristics of the data were evaluated using both visual methods (histograms and Q–Q plots) and analytical tests (Kolmogorov–Smirnov and Shapiro–Wilk tests). Because most variables did not meet the assumptions of normal distribution, non-parametric statistical methods were applied.

Between-group comparisons based on hand dominance (right-handed *vs* left-handed) were conducted using the Mann–Whitney U test. Within-group comparisons of Functional Head Impulse Test scores obtained during rightward and leftward head movements were analysed using the Wilcoxon signed ranks test.

Numerical variables were presented as mean ± standard deviation, and minimum and maximum values, while categorical variables were expressed as frequencies (*n*) and percentages. The level of statistical significance was set at *p* < 0.05.

Effect sizes (*r*) were calculated for Mann–Whitney U test results using the *Z* values to provide additional information about the magnitude of group differences. No formal correction for multiple comparisons was applied because of the exploratory nature of the study.

## Results

A total of 58 healthy volunteers participated in the study. Of these, 62.1 per cent were female (*n* = 36) and 37.9 per cent were male (*n* = 22), with a mean age of 30.50 ± 5.65 years (range, 21–40 years). Participants were classified into two groups based on hand dominance: Group 1 consisted of right-handed individuals (*n* = 37, 63.8 per cent) and Group 2 consisted of left-handed individuals (*n* = 21, 36.2 per cent). The mean age was 31.51 ± 6.24 years in Group 1 and 28.71 ± 3.95 years in Group 2. The demographic characteristics of the participants are summarised in Table 1.

**Table 1.** Demographic characteristics of individuals Table 1 long description.

Population	*n* (%)	Female (*n*)	Male (*n*)	Age (mean ± SD; years)
Group 1	37 (63.8)	26	11	31.51 ± 6.24
Group 2	21 (36.2)	10	11	28.71 ± 3.95
Total	58 (100)	36	22	30.50 ± 5.65

SD = standard deviation

Comparisons between Group 1 (right-handed) and Group 2 (left-handed) based on head movement direction and acceleration levels in the functional head impulse test revealed statistically significant differences only during leftward head movements. At an acceleration level of 4000°/s^2^, the difference between the groups was statistically significant (*p* = 0.035). Similarly, a significant group difference was observed at 6000°/s^2^ (*p* = 0.015). In addition, the total reading score for leftward head movements was significantly higher in Group 1 compared with Group 2 (*p* = 0.006). These findings indicate that right-handed individuals demonstrated better functional vestibulo-ocular performance than left-handed individuals during leftward head impulses, particularly at higher acceleration levels (Table 2).

**Table 2.** Functional Head Impulse Test scores according to hand dominance Table 2 long description.

	Functional Head Impulse Test score					
	Group 1		Group 2			
Parameter	Mean ± SD	Minimum–maximum	Mean ± SD	Minimum–maximum	*r*	*p* value
Head acceleration, right (°/s^2^)						
– 4000	92.89 ± 10.59	67−100	94.71 ± 9.58	67−100	0.043	0.745
– 5000	89.86 ± 11.19	60−100	89.06 ± 11.50	60−100	0.023	0.860
– 6000	87.46 ± 16.51	50−100	83.14 ± 11.70	50−100	0.192	0.144
Total reading score (%)	90.22 ± 10.48	63−100	89.45 ± 7.06	75−100	0.129	0.324
Head acceleration, left (°/s^2^)						
– 4000	95.57 ± 10.48	60−100	93.70 ± 9.21	67−100	0.276	0.035*
– 5000	91.81 ± 13.32	63−100	86.48 ± 14.29	50−100	0.220	0.094
– 6000	88.95 ± 17.97	40−100	83.46 ± 13.30	40−100	0.321	0.015*
Total reading score (%)	92.41 ± 11.75	63−100	87.87 ± 9.90	57−100	0.364	0.006*

SD = standard deviation; *r* = effect size; *: statistically significant p-value

### The effect of hand dominance on scores

According to the Functional Head Impulse Test data analysed based on hand dominance, no statistically significant differences were observed between right- and left-handed individuals during rightward head movements at any acceleration level (*p* > 0.05). Although right-handed participants showed slightly higher scores at 6000°/s^2^, this difference did not reach statistical significance (*p* = 0.144). The effect sizes calculated for rightward head movements were very small to small across all acceleration levels and for the total reading scores, indicating minimal practical differences between the groups.

In contrast, statistically significant differences were observed during leftward head movements. At 4000°/s^2^, right-handed individuals achieved significantly higher mean scores compared with left-handed individuals (*p* = 0.035, *r* = 0.276). Similarly, at 6000°/s^2^, right-handed participants again demonstrated superior performance (*p* = 0.015, *r* = 0.321). The total reading score for leftward head movements was also significantly higher in the right-handed group (*p* = 0.006, *r* = 0.364), indicating a moderate effect size. Although the difference at 5000°/s^2^ did not reach statistical significance (*p* = 0.094), a trend towards better performance in right-handed individuals was observed.

Overall, these findings suggest that hand dominance influences functional vestibulo-ocular performance primarily during leftward head impulses, with right-handed individuals demonstrating superior performance at higher acceleration levels.

#### The effect of head movement direction on scores

The effect of head movement direction on Functional Head Impulse Test performance was evaluated by comparing rightward and leftward head impulse scores within each hand dominance group. In the left-handed group (*n* = 21), no statistically significant differences were observed between rightward and leftward head movements at any acceleration level or in the total reading score (*p* > 0.05). Similarly, in the right-handed group (*n* = 37), Functional Head Impulse Test scores did not differ significantly between head movement directions across all acceleration levels and for the total scores (*p* > 0.05), as shown in Table 3.

**Table 3.** Effect of head movement direction on Functional Head Impulse Test scores Table 3 long description.

Parameter	Group 1 score (mean ± SD)	*p* value	Group 2 score (mean ± SD)	*p* value
Head acceleration 4000°/s^2^				
– Right head movement direction	92.89 ± 10.59	0.12	94.71 ± 9.58	0.58
– Left head movement direction	95.57 ± 10.48		93.70 ± 9.21	
Head acceleration 5000°/s^2^				
– Right head movement direction	89.86 ± 11.19	0.23	89.06 ± 11.50	0.26
– Left head movement direction	91.81 ± 13.32		86.48 ± 14.29	
Head acceleration 6000°/s^2^				
– Right head movement direction	87.46 ± 16.51	0.65	83.14 ± 11.70	0.72
– Left head movement direction	88.95 ± 17.97		83.46 ± 13.30	
Total reading score (%)				
– Right head movement direction	90.22 ± 10.48	0.4	89.45 ± 7.06	0.21
– Left head movement direction	92.41 ± 11.75		87.87 ± 9.90	

SD = standard deviation

When the analysis was extended to the pooled sample, with all 58 participants compared regardless of hand dominance, no significant differences were observed between rightward and leftward head movements at any acceleration level (4000°/s^2^: *Z* = −1.025, *p* = 0.305, |*r*| = 0.135; 5000°/s^2^: *Z* = −0.202, *p* = 0.840, |*r*| = 0.027; 6000°/s^2^: *Z* = −0.782, *p* = 0.434, |*r*| = 0.103) or for the total reading score (*Z* = −0.451, *p* = 0.652, |*r*| = 0.059). Mean group differences between directions did not exceed 1.5 percentage points and effect sizes were uniformly small. These pooled results indicate that, in the absence of stratification by hand dominance, the lateralised performance pattern observed in the handedness-based sub-group analysis is not detectable.

These findings indicate that head movement direction alone did not significantly affect vestibulo-ocular reflex performance in either group. Instead, the observed performance differences were primarily associated with hand dominance rather than with the direction of head impulses.

## Discussion

In this study, the effect of hand dominance on vestibulo-ocular reflex performance during head movements was evaluated using the Functional Head Impulse Test. The results demonstrate that right-handed individuals exhibit significantly higher Functional Head Impulse Test scores during leftward head movements, particularly at higher acceleration levels (4000 and 6000°/s^2^) compared with left-handed individuals. In contrast, no significant differences were observed between the groups during rightward head movements. These findings suggest that hand dominance may influence functional vestibular performance in a direction-specific manner, potentially reflecting hemispheric lateralisation in vestibular processing.^11^^–^^13^

Although the group difference at 5000°/s^2^ did not reach statistical significance (*p* = 0.094), the directional pattern paralleled that observed at 4000 and 6000°/s^2^, with left-handed participants showing visibly lower Functional Head Impulse Test scores than right-handed participants (86.48 ± 14.29 *vs* 91.81 ± 13.32). The mean difference (5.3 percentage points) and effect size (*r* = 0.220) were comparable in magnitude to those at the significant acceleration levels. The non-significant result is therefore most plausibly attributable to greater within-group variability at this acceleration level combined with the modest size of the left-handed group (*n* = 21), which together reduced statistical power, rather than to a genuine absence of effect.

The observed superiority of right-handed individuals during leftward head movements may be explained by hemispheric asymmetry in vestibular processing. Neuroimaging studies have consistently demonstrated that vestibular information is predominantly processed in the right cerebral hemisphere, independent of handedness.^11^^,^^12^ Meta-analytical findings further support the presence of a lateralised vestibular cortical network, with the right hemisphere playing a dominant role in spatial orientation and self-motion perception.^13^ In addition to functional asymmetry, structural connectivity studies have revealed lateralised organisation within the multimodal vestibular cortical network, highlighting stronger right-hemispheric connections in regions involved in vestibular integration.^15^

The clinical applications of the Functional Head Impulse Test are increasing because it provides a fast and practical method for assessing functional vestibulo-ocular performance under dynamic, real-life conditions.^16^ Unlike conventional vestibular tests, the Functional Head Impulse Test offers insight into the visual consequences of head movements by evaluating gaze stabilisation during high-acceleration impulses.

In clinical practice, considering individual neurophysiological characteristics such as hand dominance when interpreting Functional Head Impulse Test results may enhance diagnostic accuracy. This approach may help reduce the risk of false-negative or false-positive interpretations, particularly in cases where lateralised cortical processing influences vestibular performance.

In the present study, 36.2 per cent of the participants were classified as left-handed, which is higher than the prevalence reported in the general population. Large-scale meta-analyses indicate that left-handedness occurs in approximately 10.6 per cent of individuals worldwide,^8^ while studies conducted in Turkey have reported a prevalence of around 20.3 per cent.^9^ The relatively higher proportion of left-handed participants in the current sample may be attributed to volunteer-based recruitment or increased interest among left-handed individuals in participating in research studies.

Although this distribution allowed for meaningful comparisons between right- and left-handed groups, it may also limit the generalisability of the findings to the broader population, therefore the results should be interpreted with caution and future studies with larger and more representative samples are recommended to confirm the observed effects of hand dominance on functional vestibulo-ocular performance.

Various neurological studies have demonstrated that the right cerebral hemisphere plays a dominant role in processing vestibular inputs and that this functional asymmetry may be associated with hand dominance.^17^^,^^18^ Although right-handed individuals typically exhibit left-hemispheric dominance for general motor and sensory control, the right hemisphere appears to be more actively involved in the central processing of vestibular stimuli. This may help to explain why vestibular responses elicited by leftward head movements were stronger in right-handed individuals in the present study.^19^^,^^20^

This interpretation is consistent with previous findings indicating hemispheric asymmetry in the control of vestibular reflex responses.^12^ The lateralised performance differences observed in vestibulo-ocular reflex outputs are therefore likely to reflect not only peripheral reflex mechanisms but also higher-level cortical modulation of vestibular processing.^21^ In line with this perspective, our results showed significantly higher Functional Head Impulse Test performance in right-handed individuals during leftward head movements, further supporting the dominant role of the right hemisphere in vestibular functions. These findings are particularly consistent with the work of Arshad and colleagues, who provided direct behavioural evidence that handedness-related vestibular cortical dominance modulates the vestibulo-ocular reflex itself, demonstrating that this lower-order brainstem reflex is subject to hemispheric lateralisation rather than being purely peripheral in origin.^22^

The absence of significant differences in Functional Head Impulse Test scores during rightward head movements suggests that the influence of hand dominance on vestibulo-ocular performance may be direction-specific rather than generalised. Neuroimaging studies have shown that hemispheric activation patterns vary depending on the direction of vestibular stimulation, with leftward and rightward head movements engaging cortical networks differently.^11^^,^^13^ In particular, vestibular stimuli that preferentially activate the right hemisphere may produce more pronounced functional effects, especially in right-handed individuals.

Functional magnetic resonance imaging studies have also demonstrated that cortical and subcortical vestibular responses differ according to stimulus characteristics, reflecting complex interactions between sensory input and hemispheric specialisation, therefore the lack of significant group differences during rightward head movements in the present study may be related to more balanced bilateral cortical activation under these conditions, resulting in comparable vestibulo-ocular reflex performance across hand dominance groups.

The lack of significant differences between rightward and leftward head movements within each hand dominance group suggests a relatively symmetrical functional organisation of the vestibulo-ocular reflex. The vestibulo-ocular reflex is known to operate through bilateral and highly integrated brainstem and cortical pathways, allowing for co-ordinated eye movements regardless of head movement direction.^3^ This bilateral organisation supports stable gaze during everyday activities that involve frequent and multidirectional head movements.

Previous research has also indicated that, despite hemispheric asymmetries in cortical vestibular processing, basic reflexive vestibulo-ocular responses tend to remain functionally balanced under normal conditions,^23^^,^^24^ therefore the absence of directional differences within each group in the present study is consistent with the robust and symmetrical nature of the vestibulo-ocular reflex system.

From a clinical perspective, these findings suggest that head movement direction alone is unlikely to bias Functional Head Impulse Test performance in healthy individuals. Instead, individual neurophysiological factors such as hand dominance and hemispheric specialisation may play a more prominent role in shaping subtle variations in functional vestibular responses.

This interpretation is reinforced by the pooled analysis of all 58 participants, in which no significant difference between rightward and leftward head movements emerged at any acceleration level. The directional effect therefore becomes visible only when participants are stratified by hand dominance, providing further evidence that handedness is a relevant modulator of functional vestibulo-ocular performance and that aggregating groups without considering lateralisation can obscure clinically meaningful patterns.

In addition to hemispheric lateralisation, several cognitive and sensory factors may influence Functional Head Impulse Test performance. Vestibular processing has been shown to interact with visuospatial attention, executive functions and working memory, all of which contribute to accurate gaze stabilisation during dynamic head movements.^25^ Variations in attention level, visual processing speed and task engagement may therefore affect an individual’s ability to correctly identify optotypes during the Functional Head Impulse Test.

Fatigue and motivation may also play a role, particularly in tasks that require repeated high-acceleration head impulses and rapid visual responses. Although all participants in the present study were healthy and met strict inclusion criteria, these factors were not objectively quantified. Consequently, subtle inter-individual differences in cognitive or sensory processing may have contributed to variability in Functional Head Impulse Test scores.

Future studies incorporating objective measures of attention, visual function and cognitive load may help to further clarify the extent to which these factors interact with vestibular performance and hand dominance.

Several limitations of the present study should be acknowledged. Firstly, the sample size was relatively modest, particularly for the left-handed group, which may have limited the statistical power to detect smaller effects of hand dominance on Functional Head Impulse Test performance. Although significant differences were observed for leftward head movements, larger and more balanced samples are needed to confirm the robustness of these findings.

Secondly, cortical activation patterns were not directly assessed in this study. While the interpretation of the results was supported by existing neuroimaging literature on hemispheric vestibular lateralisation,^11^^,^^13^^,^^14^ the absence of functional or structural brain imaging data prevents direct conclusions about the neural mechanisms underlying the observed performance differences.

A further methodological consideration concerns the potential influence of the examiner’s handedness because all head impulses were delivered manually by a single experienced right-handed audiologist. Although small inter-trial variations in the force or timing of manually delivered impulses are possible, the Functional Head Impulse Test software provided real-time feedback on whether each impulse fell within the targeted acceleration range and allowed trials with inadequate stimulus profiles, together with their associated responses, to be excluded from the analysis, ensuring that the retained impulses were as controlled and symmetrical as possible in both directions. Nevertheless, examiner handedness was not systematically varied in the present design and future studies involving examiners with both hand dominance profiles would help to formally isolate any such effect.

Finally, the study focused exclusively on semicircular canal-mediated vestibulo-ocular reflex responses. Future research should also examine whether hand dominance influences other vestibular functions, such as otolith-mediated reflexes or postural control mechanisms. Combining the Functional Head Impulse Test with neuroimaging techniques and comprehensive vestibular assessments may provide a more detailed understanding of how hemispheric lateralisation shapes vestibular processing.

- The Functional Head Impulse Test is a validated tool for assessing the dynamic vestibulo-ocular reflex
- Although vestibular processing exhibits hemispheric lateralisation, the role of individual factors such as hand dominance in modulating functional vestibular test outcomes remains unclear
- Hand dominance significantly influences Functional Head Impulse Test performance during leftward head impulses, with right-handed individuals achieving higher scores at high acceleration levels (4000 and 6000°/s^2^)
- Incorporating hand dominance into the interpretation of Functional Head Impulse Test results may enhance the individualisation and clinical accuracy of vestibular assessments

## Conclusions

This study demonstrated that hand dominance is associated with functional vestibulo-ocular performance during leftward head movements, with right-handed individuals showing superior Functional Head Impulse Test scores at higher acceleration levels. These findings suggest that hemispheric lateralisation may influence the functional expression of vestibular reflexes under dynamic conditions. Importantly, head movement direction alone did not significantly affect Functional Head Impulse Test performance within each group, indicating that the observed differences were primarily related to individual neurophysiological characteristics rather than directional asymmetry.

From a clinical perspective, these results highlight the potential value of considering hand dominance when interpreting Functional Head Impulse Test outcomes in healthy individuals. Incorporating lateralisation-related factors into vestibular assessment may contribute to more individualised and accurate diagnostic interpretations. Moreover, awareness of such subtle performance differences may support the development of personalised vestibular rehabilitation strategies tailored to individual neurophysiological profiles. Further multidisciplinary studies integrating functional testing with neuroimaging approaches are warranted to clarify the cortical mechanisms underlying these effects and to extend these findings to clinical populations.

## Data Availability

Data or code available on reasonable request from authors.

## Table 1 Long description

Participant demographics are summarized for two groups and the overall sample, including group size and percent of the sample, counts of females and males, and average age with variability. Group 1 included 37 participants, representing 63.8 percent of the sample, with 26 females and 11 males. Group 2 included 21 participants, representing 36.2 percent, with 10 females and 11 males. Mean age was higher in Group 1 at 31.51 years with a standard deviation of 6.24, compared with 28.71 years with a standard deviation of 3.95 in Group 2. Overall, the sample totaled 58 participants with 36 females and 22 males, and a mean age of 30.50 years with a standard deviation of 5.65. Sex distribution differs by group, with Group 1 skewing female while Group 2 is nearly balanced. Age comparisons are based on group averages and do not show the full age distribution or statistical significance.

Navigate back to Table 1.

## Table 2 Long description

Functional Head Impulse Test reading scores are reported for two groups at three head-acceleration levels on the right and left sides, plus a total reading score for each side. For right-side accelerations, group means are close at 4000, 5000, and 6000, and the right total reading score is also similar; all right-side p values indicate no clear group difference. For left-side accelerations, Group 1 has higher mean scores than Group 2 at 4000 and 6000, and these comparisons have p values marked as statistically significant. Left-side results at 5000 show a smaller difference and are not marked significant. The left total reading score is higher in Group 1 than Group 2 and is marked significant. Effect sizes range from very small on the right to small-to-moderate on the left, so statistically significant findings are concentrated on the left side and should be interpreted alongside the size of the differences and score ranges.

Navigate back to Table 2.

## Table 3 Long description

Functional Head Impulse Test reading scores are reported as mean and standard deviation for two groups, comparing right versus left head movement at head accelerations of 4000, 5000, and 6000 degrees per second squared, plus a total reading score percent. At 4000, Group 1 averaged 92.89 for right and 95.57 for left, while Group 2 averaged 94.71 for right and 93.70 for left; the reported p values were 0.12 and 0.58. At 5000, Group 1 averaged 89.86 right and 91.81 left, and Group 2 averaged 89.06 right and 86.48 left; p values were 0.23 and 0.26. At 6000, Group 1 averaged 87.46 right and 88.95 left, and Group 2 averaged 83.14 right and 83.46 left; p values were 0.65 and 0.72. For total reading score percent, Group 1 averaged 90.22 right and 92.41 left, and Group 2 averaged 89.45 right and 87.87 left; p values were 0.40 and 0.21. Across all conditions, right and left movement scores are close within each group and none of the reported p values suggest a clear direction effect; variability is moderate to large, especially at 6000 in Group 1.

Navigate back to Table 3.
